# Prognostic performance of three lymph node staging schemes for patients with Siewert type II adenocarcinoma of esophagogastric junction

**DOI:** 10.1038/s41598-017-09625-z

**Published:** 2017-08-31

**Authors:** Jinming Xu, Jinlin Cao, Luming Wang, Zhitian Wang, Yiqing Wang, Yihua Wu, Wang Lv, Jian Hu

**Affiliations:** 1 Department of Thoracic Surgery, the First Affiliated Hospital, Zhejiang University School of Medicine, Hangzhou, 310003 China; 2 Department of Toxicology, Zhejiang University School of Public Health, Hangzhou, 310058 China

## Abstract

The prognostic performance of different lymph node staging schemes for adenocarcinoma of esophagogastric junction (AEG) remains controversial. The objective of the present study was to compare the prognostic efficacy of the number of lymph node metastases (LNMs), the positive lymph node ratio (LNR) and the log odds of positive lymph nodes (LODDS). Patients diagnosed with Siewert type II AEG were included from the Surveillance, Epidemiology, and End Results database. Harrell’s C-index statistic, Schemper’s proportion of explained variation (PEV), the Akaike information criterion (AIC) and restricted cubic spine analyses were adopted to assess the predictive accuracy of LNM, LNR and LODDS. A total of 1302 patients with post-surgery Siewert type II AEG were included. LNM, LNR and LODDS all showed significant prognostic value in the multivariate Cox regression analyses. LODDS performed higher predictive accuracy than LNM and LNR, with relatively higher C-index, higher Schemper’s PEV value and lower AIC value. For patients with no nodes involved, LODDS still performed significantly discriminatory utility. LODDS showed more accurate prognostic performance than LNM and LNR for post-surgery Siewert type II AEG, and it could help to detect survival heterogeneity for patients with no positive lymph nodes involved.

## Introduction

The incidence of adenocarcinoma of esophagogastric junction (AEG) has increased dramatically in both Western and Asian countries over the past several decades^1–4^, which might be caused by the increasing trend of gastroesophageal reflux disease, obesity and smoking^5–8^. The definition and classification of AEG remains controversial, and the Siewert’s classification^9, 10^ was commonly used and adopted by the seventh edition of Union for International Cancer Control (UICC) and American Joint Committee on Cancer (AJCC) tumor-node-metastasis (TNM) staging system^11^. AEG was defined as adenocarcinoma whose epicenter is in the distal thoracic esophagus, esophagogastric junction (EGJ), or within the proximal 5 cm of the stomach (cardia) that extend into the EGJ or distal thoracic esophagus and Siewert type II AEG arises from the cardiac epithelium^11^. AEG was staged identically to the staging criteria of esophageal cancer in AJCC 7th edition and the absolute number of lymph node metastases (LNMs) is currently used for the N stage. However, it has been reported that the current nodal staging criteria could be influenced by total number of lymph node retrieved and might cause stage migration^12, 13^.

Recently, several different lymph node staging schemes were proposed for esophageal cancer^14, 15^ and gastric cancer^16–18^, including the number of LNMs, the positive lymph node ratio (LNR) and the log odds of positive lymph nodes (LODDS). LNR was defined as the ratio of the number of metastatic lymph nodes to the total number of examined lymph nodes^12^ and LODDS was defined as the natural logarithm of the ratio of the probability of a lymph node being positive to the probability of a lymph node being negative when a single lymph node is retrieved^19^. However, the comparison of three lymph node staging schemes has not been evaluated specifically for Siewert type II AEG, and it’s important to find the optimal prognostic indicator to provided evidence and improvement for the current TNM classification system. Thus, the aim of this study was to compare the prognostic efficacy of LNM, LNR and LODDS in patients with post-surgery Siewert type II AEG using the Surveillance, Epidemiology, and End Results (SEER) database of 18 registries.

## Materials and Methods

### Study population

Patients were selected using the SEER*Stat software (version 8.3.2) from the latest version of SEER database (SEER 18, 1973–2013), which was released in April 2016 and based on the November 2015 submission^20^. Patients were eligible for inclusion if they were at least 18 years old and diagnosed with microscopically confirmed primary AEG with no distant metastasis from 1988 to 2013. The histology types were restricted according to the International Classification of Disease-Oncology-3^rd^ edition (ICD-O-3), with codes of 8050, 8140–8147, 8160–8162, 8180–8221, 8250–8507, 8514–8551, 8571–8574, 8576, and 8940–8941 and tumor site codes of 160–162. The type of follow-up expected was restricted to ‘active follow-up’, and the survival time should not be less than two months after the operation. A schema discriminator ‘CS Site-Specific Factor 25’ for distinguishing EGJ and stomach was used to distinguish AEG from gastric cancer, with codes of 020, 040, 060 and 982. The 5-year overall survival (OS) was selected as the outcome of interests for this study. Survival time was defined as the time between diagnosis and the death, the last contact or the cutoff date of December 31, 2013. The detailed selection codes for SEER database were shown in the Supplementary File 1.

A second round selection was conducted, and the detailed exclusion process was shown in Supplementary Figure 1. The tumor extension information of AEG was not available before the year of 2004, and patients diagnosed with AEG after 2008 were excluded in order to ensure the 5-year follow-up. Thus, patients diagnosed between 2004 and 2008 were finally included. In addition, those who didn’t receive surgery, diagnosed with exfoliative cytology results, with incomplete survival dates and distant metastasis were excluded. Tumors with mixed histopathologic types were also excluded, because the mixed type was classified as squamous cell carcinoma according to the seventh edition UICC/AJCC TNM staging system. Although the SEER database didn’t provide the detailed information of Siewert type classification for AEG, the combined selection terminology of “Primary Site” encoded 160 (Cardia) and “CS site-specific factor 25” encoded 982 (EsophagusGEJunction) allowed us to obtain the Siewert type II AEG^21^. No institutional review board approval was declared because the SEER is a publicly available database.

### Node staging schemes

LNM was defined identically to lymph node staging classification of the seventh edition of UICC/AJCC TNM staging system^22^, which was based on the number of positive lymph nodes examined: 0 (LNM-N0), 1–2 (LNM-N1), 3–6 (LNM-N2), ≥7 (LNM-N3). LNR was defined as the ratio of the number of positive lymph nodes to the total number of retrieved lymph nodes, which ranged from 0 to 1. LODDS value was calculated by the formula log_e_[(pN + 0.5)/(nN + 0.5)]^23^, where pN is the number of positive lymph nodes and nN is the number of negative lymph nodes and nN was calculated by subtracting pN from the total examined lymph nodes. A value of 0.5 was added to pN and nN to avoid singularity caused by null observations^19^.

### Statistical analysis

Spearman rank tests and scatter plots were adopted to assess the relationship between LODDS and LNM or LNR and to elucidate the distribution characteristics. Univarite analysis was first conducted to evaluate the prognostic performance of these lymph node schemes, and then multivariate analysis was performed based on statically significant factors in the univarite analysis. Then, restricted cubic splines were plotted to further display the association between log hazard ratio and LNM, LNR and LODDS^24^. The optimal cut-off values for LNR and LODDS were determined by X-tile software (http://www.tissuearray.org/rimmlab) and by the minimal *P* value approach^25^. The discrimination efficacy of the prognostic schemes was assessed by Harrell’s C-index, which ranged from 0.5 (denotes random splitting) to 1 (perfect prediction)^26^. Besides, the bootstrap technique with 1000 repetitions was used for validation and to calculate the 95% confidence interval (CI). Schemper’s proportion of explained variation (PEV), generally known as R^2^, could be used to measure predictive accuracy and explain variation for a specific predictor or model^27^. And a higher PEV value represented better prognostic accuracy. In addition, Akaike information criterion (AIC) was also applied to further evaluate the predictive efficacy for different lymph node staging schemes, and smaller AIC values represent more accurate prognostic stratification^28^.

All analyses were conducted using the SPSS 19.0 (IBM SPSS Inc. United States), Stata software (version 12.0; StatCorp, College Station, TX, USA), and R software version 3.2.2 (The R Foundation for Statistical Computing) with the Rms and Hmisc statistical packages. Statistical significance was set at *P* < 0.05 unless otherwise specified (All *P* values presented were 2-sided).

## Results

### Characteristics of patients and three lymph node staging schemes

A total of 1302 patients with resected Siewert type II AEG were finally included from the SEER database. The median survival time was 31 months and the overall 5-year survival rate was 36.25%. The characteristics and demographics of included patients were shown in Table 1. Univariate Cox regression analysis was conducted, which suggested that age, marital status, tumor size, tumor differentiation grade, T stage, LNM, LNR and LODDS were potential prognostic factors for AEG. However, gender, race, total lymph nodes retrieved and post-surgery radiation were not significantly associated with the prognosis of AEG.

**Table 1:** Demographics and tumor characteristics for patients with Siewert type II esophagogastric junction adenocarcinoma from the Surveillance, Epidemiology, and End Results databases, 2004–2008.

Variable	No of patients (N)	Percentage (N/total)	5-year survival rate	Univariate Hazard Ratio (95% CI)	*P* value^a^
Gender					0.953
Female	311	23.89%	35.37%	Reference	
Male	991	76.11%	36.53%	1.005(0.859–1.175)	0.953
Age					<0.001
<60	463	35.56%	45.14%	Reference	
60–70	384	29.49%	36.98%	1.256(1.058–1.490)	0.009
>70	455	34.95%	26.59%	1.759(1.501–2.062)	<0.001
Race					0.358
White	1114	85.56%	35.91%	Reference	
Black	65	4.99%	32.31%	1.116(0.826–1.507)	0.475
Other	120	9.22%	40.83%	0.875(0.692–1.106)	0.263
Unknown	3	0.23%	66.67%	0.312(0.044–2.215)	0.244
Marital status					0.008
Married	895	68.74%	39.22%	Reference	
Divorced/Separated	117	8.99%	29.06%	1.259(1.002–1.580)	0.048
Single	133	10.22%	30.08%	1.230(0.990–1.528)	0.061
Widowed	127	9.75%	26.77%	1.409(1.135–1.748)	0.002
Unknown	30	2.30%	43.33%	1.002(0.635–1.583)	0.993
Tumor size (cm)					<0.001
≤3	339	26.04%	56.34%	Reference	
≤5	449	34.49%	29.62%	2.067(1.713–2.494)	<0.001
>5	391	30.03%	23.53%	2.367(1.956–2.864)	<0.001
Unknown	123	9.45%	45.53%	1.222(0.919–1.625)	0.167
Grade					<0.001
Well	67	5.15%	64.18%	Reference	
Moderately	428	32.87%	44.63%	1.463(1.007–2.124)	0.046
Poorly	733	56.30%	28.38%	2.313(1.610–3.322)	<0.001
Undifferentiated	32	2.46%	21.88%	2.656(1.576–4.476)	<0.001
Unknown	42	3.23%	54.76%	0.989(0.553–1.786)	0.970
T stage					<0.001
T1	324	24.88%	65.43%	Reference	
T2	141	10.83%	48.94%	1.717(1.300–2.269)	<0.001
T3	502	38.56%	24.70%	3.143(2.564–3.853)	<0.001
T4	334	25.65%	19.76%	3.617(2.920–4.481)	<0.001
ERROR^#^	1	0.08%			
LNM					<0.001
0	466	35.79%	61.59%	Reference	
1–2	283	21.74%	32.86%	2.327(1.915–2.828)	<0.001
3–6	258	19.82%	21.71%	3.058(2.513–3.722)	<0.001
≥7	295	22.66%	12.20%	4.066(3.369–4.907)	<0.001
LNR					<0.001
0	466	35.79%	61.59%	Reference	
≤0.125	189	14.52%	41.27%	1.868(1.494–2.337)	<0.001
≤0.425	323	24.81%	22.91%	2.834(2.351–3.416)	<0.001
≤1	324	24.88%	10.19%	4.740(3.941–5.700)	<0.001
LODDS					<0.001
≤−2.800	351	26.96%	62.11%	Reference	
≤−1.600	322	24.73%	46.89%	1.584(1.277–1.964)	<0.001
≤−0.310	304	23.35%	23.03%	2.905(2.365–3.568)	<0.001
≤4.270	325	24.96%	10.15%	4.801(3.926–5.870)	<0.001
Total lymph nodes retrieved					0.831
1–10 nodes	423	32.49%	38.06%	Reference	
11–20 nodes	513	39.40%	34.70%	1.022(0.874–1.196)	0.781
≥21 nodes	366	28.11%	36.34%	0.972(0.819–1.153)	0.744
Radiation after surgery					0.139
No	887	68.13%	38.22%	1	
Yes	415	31.87%	32.05%	1.111(0.967–1.277)	0.139

^a^Univariate Cox regression analysis.

^#^Obsolete data retained V0200, and the detailed polyp information was not available.

^−^Not available.

LNM: lymph node metastasis.

LNR: positive lymph node ratio.

LODDS: log odds of positive lymph node.

**Figure 1:**
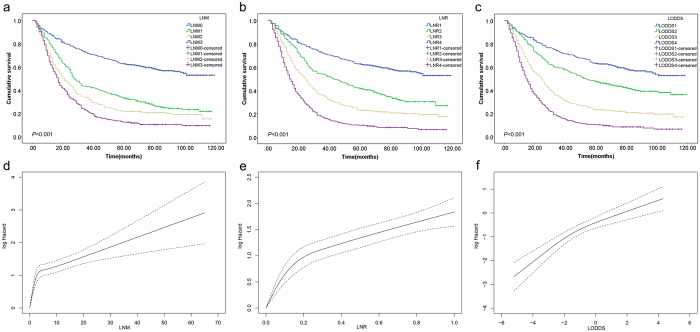
Kaplan-Meier (KM) survival curves and log hazard ratio cubic spline analyses for patients identified from the SEER database by different lymph node staging schemes. (**a**) KM curves by LNM scheme (log rank *P* < 0.001) (**b**) KM curves by LNR scheme (log rank *P* < 0.001). (**c**) KM curves by LODDS scheme (log rank *P* < 0.001). (**d**) log hazard ratio as function of LNM. (**e**) log hazard ratio as function of LNR. (**f**) log hazard ratio as function of LODDS.

The cut-off points of LNR and LODDS were determined by the X-tile software, which could explore the minimal *P* value. According to the X-tile analysis results (Supplementary Figure 2a and b), LNR was classified into LNR1 (value 0), LNR2 (0 < LNR2 ≤ 0.125) LNR3 (0.125 < LNR3 ≤ 0.425) and LNR4 (0.425 < LNR4 ≤ 1.000), and LODDS was classified into LODDS1 (−5.20 ≤ LODDS1 ≤ −2.800), LODDS2 (−2.800 < LODDS2 ≤ −1.600), LODDS3 (−1.600 < LODDS3 ≤ −0.310) and LODDS4 (−0.310 < LODDS4 ≤ 4.270). As shown in Table 1, the 5-year OS rates for LNR scheme were 61.59% (LNR1), 41.27% (LNR2), 22.91% (LNR3) and 10.19% (LNR4), respectively (*P* < 0.001). For LODDS scheme, the 5-year OS rates were 62.11% (LODDS1), 46.89% (LODDS2), 23.03% (LODDS3), and 10.15% (LODDS4), respectively (*P* < 0.001). The Kaplan-Meier survival curves and log hazard ratio cubic spline analyses results according to three different lymph node staging schemes were shown in Fig. 1. The three lymph node schemes all showed significant discrimination efficacy and good prognostic performance for patients with resected Siewert type II AEG (log rank *P* < 0.001) (Fig. 1a,b and c). In addition, the cubic spline of log hazard ratios revealed non-linear increasing trend of death risk as LNM, LNR or LODDS increased (Fig. 1d,e and f). Due to the relatively small sample size for patients with no positive lymph nodes involved (N = 466) and all lymph nodes involved (N = 42), the cubic spline was not conducted in these subgroups.

**Figure 2:**
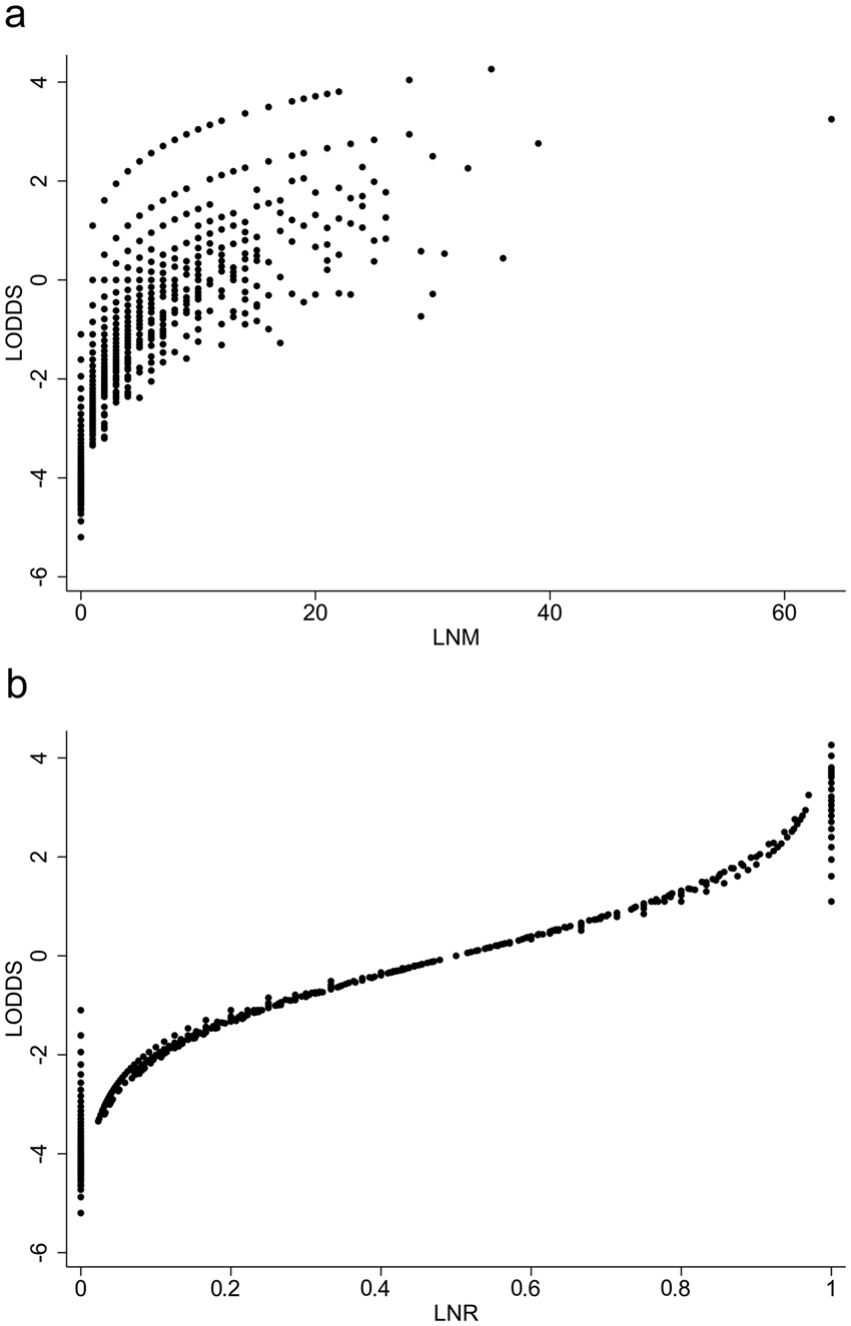
Distribution characteristics of (**a**) log odds of positive lymph nodes (LODDS) and number of lymph nodes metastasis (LNM) (*r* = 0.735, Spearman rank test *P* < 0.001) and (**b**) LODDS and positive lymph node ratio (LNR) (*r* = 0.943, Spearman rank test *P* < 0.001).

**Figure 3:**
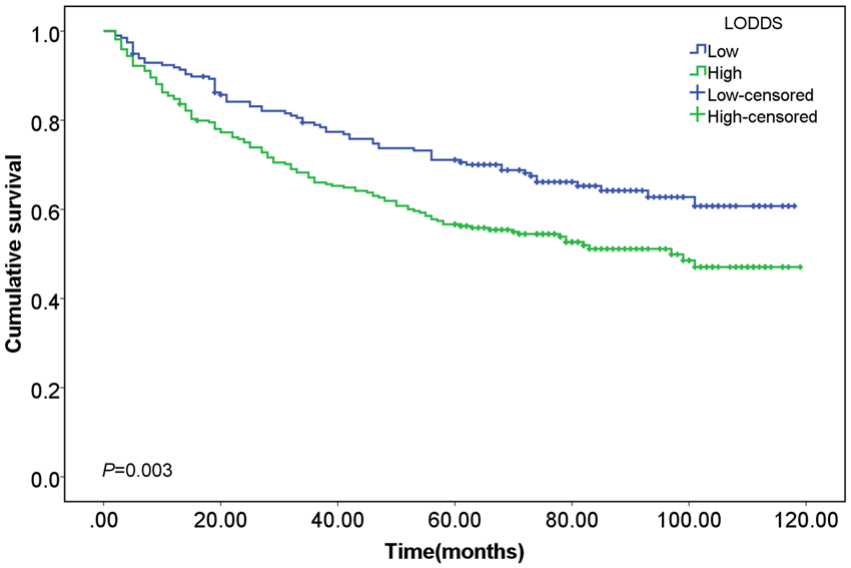
Kaplan-Meier survival curves for patients with no positive lymph nodes involved (log rank *P* = 0.001, and the cut-off intervals for LODDS in this group were −5.20 to −3.37 and −3.37 to −1.10, determined by the x-tile software).

As shown in Fig. 2, the scatter plots suggested significant positive association between LODDS and LNM (Spearman rank test *P* < 0.001) and between LODDS and LNR (Spearman rank test *P* < 0.001), and LODDS had a higher coefficient correlation with LNR than with LNM (*r* = 0.943 versus *r* = 0.735), which indicated more accurate fitness between LODDS and LNR. However, for patients with no positive lymph nodes involved (LNR0) and all lymph nodes involved (LNR1), the LODDS value scattered out. In order to further explore the prognostic value of LODDS in these patients with relatively small sample size, we recalculated the cut-off value of LODDS and classified it into high and low categories by X-tile software, as shown in the Supplementary Figure 2c. For patients with no positive lymph nodes involved, the high and low intervals were −3.37 to −1.10 and −5.20 to −3.37, respectively. Then, Kaplan-Meier survival curves were conducted and the LODDS could distinguish the survival heterogeneity for patients with no positive lymph nodes involved (Fig. 3, ﻿log rank *P* = 0.003). However, the prognostic value of LODDS for patients with all lymph nodes involved was not further evaluated due to the small sample size (n = 42).

### Comparison of the prognostic performance for three lymph node staging schemes

Multivariate Cox regression analyses were further conducted to assess the lymph node staging schemes, as shown in Table 2. LNM, LNR and LODDS were all independent prognostic factors for post-surgery Siewert type II AEG (*P* < 0.001), and other independent prognostic factors included age, marital status, and T stage. According to the comparison results of the three lymph node schemes in Table 3, LODDS had the highest C-index (95% CI) of 0.673(0.654–0.692), and LODDS and LNR both performed significantly better prognostic efficacy than the LNM scheme, with *P*-values of 0.045 and 0.025, respectively. Although the comparison between LODDS and LNR was not statistically significant, the Schemper’s PEV and AIC of LODDS were 15.90% and 1502.119, which indicated that LODDS scheme could explain variation better and showed superior predictive accuracy than LNM and LNR schemes (Table 3). Then, we combined the three node staging schemes with T stages to compare the modified T-LODDS-M and T-LNR-M staging system to the AJCC 7th TNM staging system. As shown in Table 3, the T-LODDS system still performed the highest C-index of 0.683(0.664–0.702), and T-LODDS (*P* = 0.005) and T-LNR (*P* = 0.015) systems were still both significantly better than the traditional TNM staging system. In order to further evaluate the stability of these prognostic factors, combined models with significant prognostic factors in the multivariate analysis were constructed, including age, marital status, T stage and the three lymph node staging schemes. Harrell’s C-index analysis, Schemper’s PEV and AIC analyses were conducted and the results also indicated the model with LODDS performed the best predictive accuracy, with the largest C-index of 0.707(0.689–0.725), the largest Schemper’s PEV of 24.90% and the smallest AIC value of 1323.464 among three models with different node staging schemes.

**Table 2:** Multivariate Cox regression analysis of prognostic factors with different lymph node classifications for Siewert type II esophogastric junction adenocarcinoma.

Variable	LNM classification		LNR classification		LODDS classification	
	HR (95% CI)	*P* value	HR (95% CI)	*P* value	HR (95% CI)	*P* value
Age		<0.001		<0.001		<0.001
<60	Reference		Reference		Reference	
60–70	1.493(1.252–1.781)	<0.001	1.471(1.234–1.754)	<0.001	1.465(1.229–1.746)	<0.001
>70	2.303(1.940–2.735)	<0.001	2.286(1.928–2.710)	<0.001	2.308(1.947–2.736)	<0.001
Marital status		0.033		0.007		0.008
Married	Reference		Reference		Reference	
Divorced/Separated	1.354(1.074–1.706)	0.010	1.450(1.150–1.829)	0.002	1.460(1.158–1.841)	0.001
Single	1.276(1.021–1.595)	0.032	1.283(1.026–1.605)	0.029	1.270(1.015–1.589)	0.037
Widowed	1.047(0.837–1.310)	0.687	1.109(0.886–1.388)	0.367	1.125(0.899–1.408)	0.304
Unknown	0.885(0.559–1.404)	0.605	0.869(0.549–1.378)	0.551	0.901(0.568–1.430)	0.659
Tumor size		0.374		0.117		0.182
≤3	Reference		Reference		Reference	
≤5	1.124(0.910–1.388)	0.279	1.148(0.929–1.418)	0.201	1.140(0.922–1.410)	0.225
>5	0.998(0.797–1.249)	0.985	0.958(0.764–1.200)	0.708	0.961(0.767–1.205)	0.731
unknown	1.148(0.856–1.538)	0.356	1.148(0.857–1.539)	0.355	1.086(0.812–1.454)	0.578
Grade		0.171		0.133		0.105
well	Reference		Reference		Reference	
moderately	1.001(0.685–1.462)	0.998	1.034(0.708–1.511)	0.861	1.029(0.704–1.503)	0.884
poorly	1.147(0.788–1.670)	0.474	1.191(0.819–1.733)	0.36	1.193(0.820–1.736)	0.355
undifferentiated	1.571(0.922–2.677)	0.096	1.649(0.967–2.810)	0.066	1.668(0.979–2.842)	0.060
unknown	1.055(0.585–1.903)	0.858	1.079(0.599–1.946)	0.8	1.069(0.593–1.927)	0.825
T stage		<0.001		<0.001		<0.001
T1	Reference		Reference		Reference	
T2	1.289(0.953–1.744)	0.099	1.259(0.931–1.704)	0.135	1.364(1.012–1.837)	0.041
T3	1.656(1.270–2.159)	<0.001	1.628(1.246–2.126)	<0.001	1.799(1.393–2.322)	<0.001
T4	1.926(1.460–2.539)	<0.001	1.853(1.404–2.447)	<0.001	2.004(1.532–2.621)	<0.001
LNM		<0.001				
0	Reference					
1–2	1.795(1.443–2.233)	<0.001				
3–6	2.346(1.862–2.957)	<0.001				
≥7	3.290(2.597–4.167)	<0.001				
LNR				<0.001		
0			Reference			
≤0.125			1.459(1.142–1.863)	0.002		
≤0.425			2.215(1.775–2.763)	<0.001		
≤1			3.933(3.125–4.950)	<0.001		
LODDS						<0.001
≤−2.800					Reference	
≤−1.600					1.403(1.126–1.748)	0.003
≤−0.310					2.343(1.871–2.934)	<0.001
≤4.270					3.984(3.161–5.021)	<0.001

LNM: lymph node metastasis.

LNR: positive lymph node ratio.

LODDS: log odds of positive lymph node.

**Table 3:** Analysis for prognostic performance of different node classifications and different models for Siewert type II esophagogastric junction adenocarcinoma.

	Harrell’s C-index (95% CI)	*P*-value1	*P*-value2	Validation^*^	Schemper’s PEV	AIC
LNM	0.653(0.634–0.672)	Ref		0.654	8.20%	1617.305
LNR	0.670(0.651–0.689)	0.045	Ref	0.670	13.90%	1533.824
LODDS	0.673(0.654–0.692)	0.025	0.405	0.672	15.90%	1502.119
T-stage + LNM	0.661(0.642–0.680)	Ref		0.660	16.10%	1488.420
T-stage + LNR	0.678(0.659–0.697)	0.015	Ref	0.677	19.00%	1444.705
T-stage + LODDS	0.683(0.664–0.702)	0.005	0.354	0.681	20.30%	1424.362
Model 1 (LNM)^a^	0.686(0.667–0.705)	Ref		0.684	20.70%	1389.390
Model 2 (LNR)^b^	0.702(0.684–0.720)	0.046	Ref	0.700	23.70%	1342.987
Model 3 (LODDS)^c^	0.707(0.689–0.725)	0.017	0.338	0.704	24.90%	1323.464

^*^By bootstrap method (B = 1000).

AIC: Akaike information criterion.

^a^A model with combined variables including age, marital status, T stage and LNM.

^b^A model with combined variables including age, marital status, T stage and LNR.

^c^A model with combined variables including age, marital status, T stage and LODDS.

LNM: lymph node metastasis.

LNR: positive lymph node ratio.

LODDS: log odds of positive lymph node.

Ref: reference category.

Continuous LNM, LNR and LODDS were included for evaluation.

*P*-value1 and *P*-value2: C-index comparisons by R language (P value = 1-pnorm(abs(r[“C × 1”] − r[“C × 2”])/(r[“S.D.”]/2))).

In order to evaluate the impact of the total number of retrieved lymph nodes on the prognostic performance of the three node staging schemes, we classified the total number of retrieved nodes as 1–10 nodes, 11–20 nodes, and equal or more than 21 nodes. According to the Harrell’s C-index analysis shown in Table 4, LODDS schemes performed the best prognostic efficacy in all subgroups, with C-index of 0.654(0.621–0.687), 0.680(0.651–0.709), and 0.690(0.656–0.724), respectively. Compared with LNM and LNR, the AIC values of LODDS in each subgroup were still the smallest, with a value of 505.314,573.338, and 430.041, respectively.

**Table 4:** Impact of total number of retrieved lymph nodes on the prognostic performance of node staging schemes for Siewert type II esophagogastric junction adenocarcinoma.

Category (No.)	1–10 nodes (n = 475)		11–20 nodes (n = 565)		≥21 nodes (n = 398)	
	C-index	AIC	C-index	AIC	C-index	AIC
LNM	0.644(0.613–0.675)	520.874	0.675(0.647–0.703)	591.415	0.688(0.654–0.722)	454.781
LNR	0.652(0.621–0.683)	509.083	0.676(0.647–0.705)	593.208	0.690(0.656–0.724)	442.109
LODDS	0.654(0.621–0.687)	505.314	0.680(0.651–0.709)	573.338	0.690(0.656–0.724)	430.041

LNM: lymph node metastasis.

LNR: positive lymph node ratio.

LODDS: log odds of positive lymph node.

Continuous LNM, LNR and LODDS were included for evaluation.

AIC: Akaike information criterion.

### Subgroup and additional analysis

T stage is an independent prognostic factor for Siewert type II AEG, and we conducted the subgroup analysis stratified by different T stages, as shown in Supplementary Table 1 and Supplementary Figure 3. The three staging systems all performed good prognostic capability in stage T1, T3 and T4 patients, while the results were not statistically significant in stage T2 patients. This might be caused by the relative small sample size of stage T2 patients (n = 141). For each T stage, LODDS still had the highest C-index, and the lowest AIC value among the three lymph node staging schemes.

In order to further analyze the prognostic value of LODDS in different T stage, N stages and LNR categories, new cut-off values of LODDS were re-calculated by X-tile analyses in different subgroups and its prognostic performance was assessed by log-rank *P*-value, as shown in Supplementary Table 2, Supplementary Figures 4, 5 and 6. LODDS had good discriminating performance in all subgroups except for stage T2 (n = 141) and stage LNR2 patients (n = 189) with relatively small sample size. In addition, linear trend χ2 score was conducted to evaluate the discriminatory ability and monotonicity of gradients, and the likelihood ratio (χ2) test was conducted to assess homogeneity ability, as shown in Supplementary Table 3. The results indicated LODDS had the best efficacy with the highest scores among three schemes.

## Discussion

The current study compared the prognostic efficacy of three lymph node staging schemes in patients with resected Siewert type II AEG from the SEER database, including LNM, LNR and LODDS. Among the three lymph node staging schemes, LODDS showed the best predictive accuracy and discriminatory utility, and consistent results were found for the multivariate model with LODDS. Compared with the traditional UICC/AJCC TNM classification, the novel schemes (LODDS and LNR) had better prognostic efficacy with higher C-index, higher Schemper’s PEV values and lower AIC values. Besides, for patients with no positive lymph nodes involved, LODDS still performed good discriminatory efficacy and revealed the survival heterogeneity among these patients, which compensated the deficiencies of LNR and conventional LNM schemes on this issue. For these people with no positive lymph nodes involved, LODDS was re-categorized as high and low levels and patients with higher LODDS values had relatively poorer prognosis.

Many studies have evaluated the prognostic value of different lymph node staging schemes^17^, and the advantages of LNR and LODDS over LNM have been validated for esophageal cancer and gastric cancer^15, 16^, as well as LNR vs LNM for AEG^29^. All these studies provided evidence for the potential promising efficacy of new lymph node staging schemes. The theoretical foundation for LNR and LODDS was the combined information of both positive and negative lymph nodes. It has been reported that LNR was a reliable indicator to improve node classification for esophageal cancer and gastric cancer with less influence by insufficient number of lymph nodes retrieved^12, 30^. Besides, the transformation of LODDS by adding a value to both the numerator and the denominator is the least biased estimator of the true log odds to avoid singularities caused by null observations^23^, which enables the rationality and more accurate discrimination for LODDS.

Although the total number of retrieved lymph nodes were not significantly associated with the prognosis of 5-year OS for the whole cohort according to the univariate Cox regression analysis results, it could impact the prognosis of patients with no positive lymph nodes involved and impact the prognostic performance of three lymph node staging schemes. The Harrell’s C-index of LNM, LNR and LODDS increased as the total number increased. LODDS was better than LNR and LNM for patients with 1–10, 11–20 and equal or more than 21 nodes retrieved, which indicated that LODDS showed more accurate prognostic performance than LNM and LNR in all subgroups and it was a relatively stable indicator. Interestingly, our study also found that marital status was an independent prognostic factor for patients with post-surgery Siewert type II AEG and marriage was proven to be a protective factor, which was consistent with the previous studies for gastric cancer^31, 32^ and esophageal cancer^33^. According to the multivariate Cox regression analysis results, the divorced or separated and single (never married) patients had relatively higher mortality rate compared with those married, thus more spiritual and social support should be given to these patients.

Although there was no statistically significant difference of C-index between LODDS and LNR, the superiority of LODDS as a novel node staging schemes for Siewert type II AEG is still recommended. Due to the unique statistical features of LODDS, it could detect the survival heterogeneity of patients with no positive lymph nodes involved and higher LODDS value predicted poorer survival of these patients. The sample size of stage N0 Siewert type II AEG patients was 466, which accounted for 35.79 percent of the total patients. Distinguishing the high-risk population with no positive lymph node involved had important clinical significance for early stage patients, and more intensive intervention, examination or treatment should be recommended for these people.

AEG was staged as an esophageal adenocarcinoma according to the seventh edition of UICC/AJCC TNM classification with a proclivity for proximal spread mainly via lymphatics in the submucosa of the esophagus^34^, however, the staging system and classification criteria for AEG remains controversial. It has been reported by Hasegawa *et al*.^35^ that the Siewert type II and III AEGs were more appropriate to be staged by the gastric cancer TNM classification. However, an eighth edition staging primer for esophageal and EJG cancer^36^ suggested that Siewert types I/II EGJ cancer should be staged as esophageal cancer, and if the cancer center is more than 2 cm distal from the EGJ, it should be staged as gastric cancer. Of note, the lymph node staging criteria were similar between esophageal cancer and gastric cancer in the seventh UICC/AJCC TNM classification^22^, with stage N0 of 0 positive node, stage N1 of 1 to 2 positive nodes, stage N2 of 3 to 6 positive nods, and stage N3 of equal or more than 7 positive nodes. Stage N3 was divided into N3a (7–15 nodes) and N3b (16 or more nodes) for gastric cancer. Thus, regardless of which system was used, the current study provided evidence for the novel lymph node staging schemes specifically in patients with Siewert type II AEG, which performed prognostic superiority over the already existing lymph node staging system and provided evidence and reference for the future staging criteria.

The prognosis of AEG is significantly associated with the extent of nodal involvement and tumor location, which could impact the lymphatic dissemination^37^, and there have been studies evaluated the lymphadenectomy approach^38^ and surgical approach for EGJ cancer^39^, as well as the multidisciplinary management for AEG^34, 40^. However, limitations should be acknowledged in the current analysis. The information of Siewert types I and III AEG was not available from the SEER database, which limited further analysis of other cancer subtypes. Besides, the detailed information of patients’ comorbidities, surgical approach and post-surgery treatment (such as chemotherapy regimens) was not provided. Patients treated with preoperative radiation therapy were excluded to reduce its impact on survival, and the impact of post-surgery radiation was not significantly associated with the 5-year OS of AEG according to the univariate analysis, while the chemotherapy information was not available and it might cause nodal down-staging.

## Conclusion

Despite these potential limitations, our study indicates that LODDS showed more accurate prognostic performance than LNM and LNR in patients with post-surgery Siewert type II AEG, and it could help to detect survival heterogeneity for patients with no positive lymph nodes involved.

### Ethical approval

All procedures performed in studies involving human participants were in accordance with the ethical standards of the institutional and/or national research committee and with the 1964 Helsinki Declaration and its later amendments or comparable ethical standards. No ethics approval was declared because the SEER is a publicly available database.

### Informed consent

We obtained permission to access SEER research data files with the reference number 11536-Nov2015. Extraction of data from the SEER database does not require informed consent.

## Electronic supplementary material

Supplementary File
