# Supplementary material for: Prognostic performance of three lymph node staging schemes for patients with Siewert type II adenocarcinoma of esophagogastric junction
Source: Sci Rep. 2017 Aug 31;7:10123. doi: 10.1038/s41598-017-09625-z (PMC5579029; doi:10.1038/s41598-017-09625-z)
Supplement: Supplementary file 1 — Supplementary File [file 41598_2017_9625_MOESM1_ESM.pdf]

Prognostic performance of three lymph node staging schemes for patients with Siewert type II adenocarcinoma of esophagogastric junction

Jinming Xu[1], Jinlin Cao[1], Luming Wang[1], Zhitian Wang[1], Yiqing Wang[1], Yihua Wu[2], Wang Lv[1], Jian Hu[1]

[1]Department of Thoracic Surgery, the First Affiliated Hospital, Zhejiang University School of Medicine, Hangzhou 310003, China; [2]Department of Toxicology, Zhejiang University School of Public Health, Hangzhou, 310058 China;

*: Corresponding to: Prof. Jian Hu, MD, PhD. Department of Thoracic Surgery, The first Affiliated Hospital, Zhejiang University School of Medicine, Hangzhou 310003, China.

E-mail: hujian_med@163.com

Tel.: +86-0571-87236847

Fax: +86-0571-86995818

Supplementary file 1. Program codes for patient selection from the Surveillance, Epidemiology, and End Results (SEER) data base.

{Race, Sex, Year Dx, Registry, County.Year of diagnosis} = '1988','1989','1990','1991','1992','1993','1994','1995','1996','1997','1998','1999','2000','2001','2002','2003','2004','2005','2006','2007', '2008','2009','2010', '2011', '2012', '2013'

AND {Race and Age (case data only).Age recode with single ages and 85+} = '18 years','19 years','20 years','21 years','22 years','23 years','24 years','25 years','26 years','27 years','28 years','29 years','30 years','31 years','32 years','33 years','34 years','35 years','36 years','37 years','38 years','39 years','40 years','41 years','42 years','43 years','44 years','45 years','46 years','47 years','48 years','49 years','50 years','51 years','52 years','53 years','54 years','55 years','56 years','57 years','58 years','59 years','60 years','61 years','62 years','63 years','64 years','65 years','66 years','67 years','68 years','69 years','70 years','71 years','72 years','73 years','74 years','75 years','76 years','77 years','78 years','79 years','80 years','81 years','82 years','83 years','84 years','85+ years'

AND {Site and Morphology.Primary Site} = 160-162

AND {Site and Morphology.Diagnostic Confirmation} = 'Microscopically confirmed'

AND {Site and Morphology.Histologic Type ICD-O-3} =8050,8140-8147,8160-8162,8180-8221,8250-8507,8514-8551,8571-8574,8576,8940-8941

AND {Extent of Disease - CS.CS site-specific factor 25 (2004+ varying by schema)} = 020, 040, 060, 982

AND {Extent of Disease - CS.Regional nodes examined (1988+)} = 1-90
AND {Extent of Disease - CS.Regional nodes positive (1988+)} = 0-90
AND {Extent of Disease - CS.CS extension (2004+)} = 100-420,450-700,800-810
AND Not{Extent of Disease - CS.CS mets at dx (2004+)} = 99
AND {Multiple Primary Fields.Sequence number} = 'One primary only'
AND {Therapy.Radiation sequence with surgery} = 'No radiation and/or cancer-directed surgery', 'Radiation after surgery'
AND {Cause of Death (COD) and Follow-up.Type of follow-up expected} = 'Active follow-up'
AND {Cause of Death (COD) and Follow-up.Survival months} = 2-491

Supplementary Figure 1. Detailed information for patient selection process.

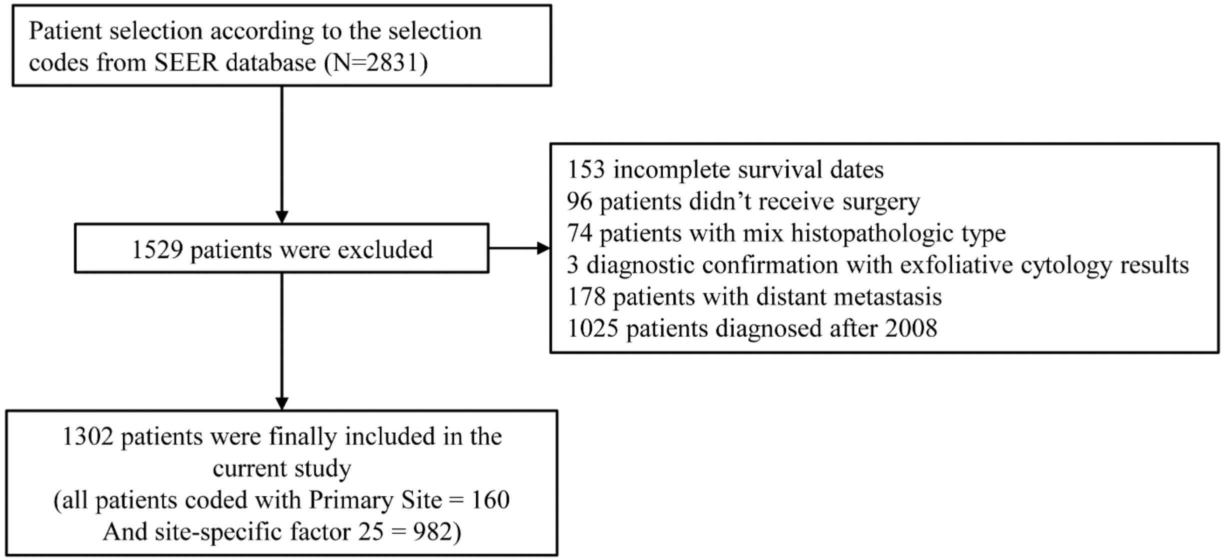

Supplementary Figure 2. Cut-off values determined by X-tile software and the minimal *P* value approach for (a) LNR (b) LODDS (c) LODDS in patients with no lymph nodes involved (n=466)

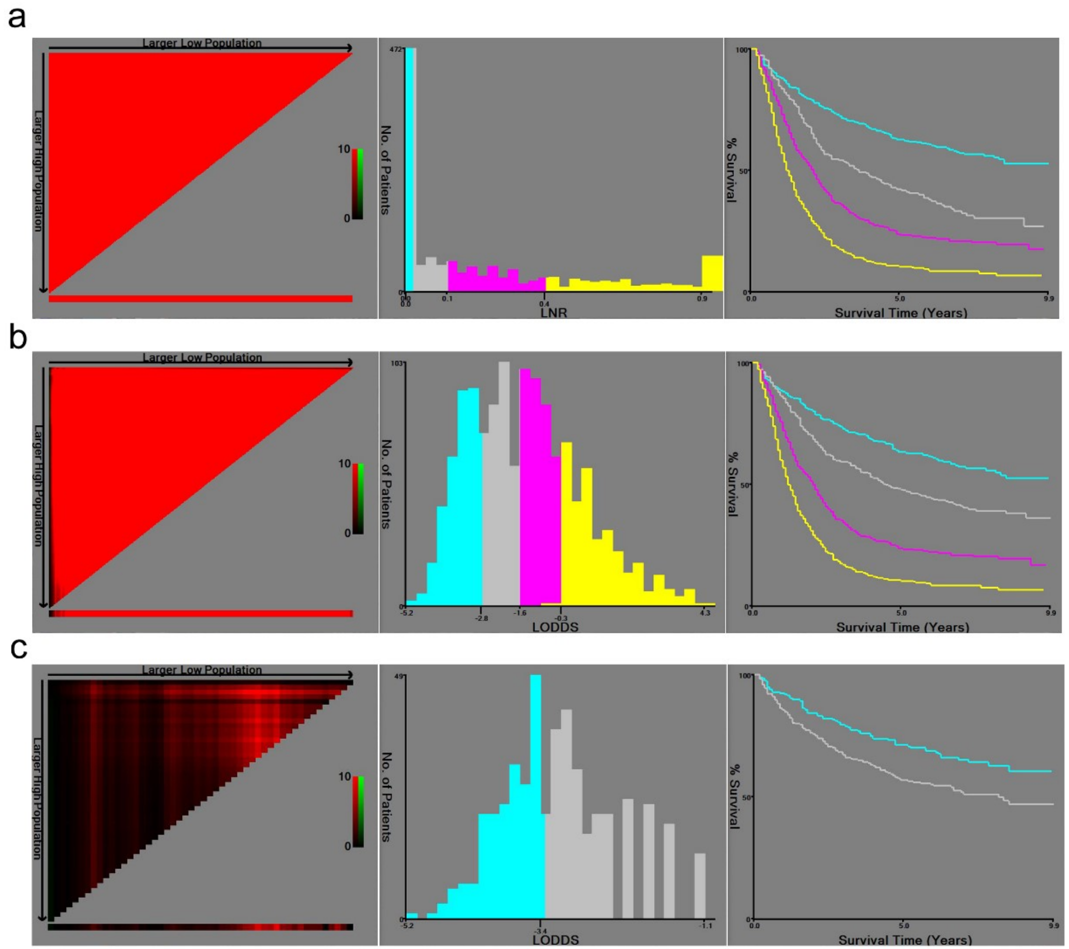

Supplementary Figure 3. Kaplan-Meier survival curves of LODDS for patients with different T stages. (a) stage T1 patients (log rank *P*<0.001), (b) stage T2 patients (log rank *P*=0.565), (c) stage T3 patients (log rank *P*<0.001), (d) stage T4 patients (log rank *P*<0.001).

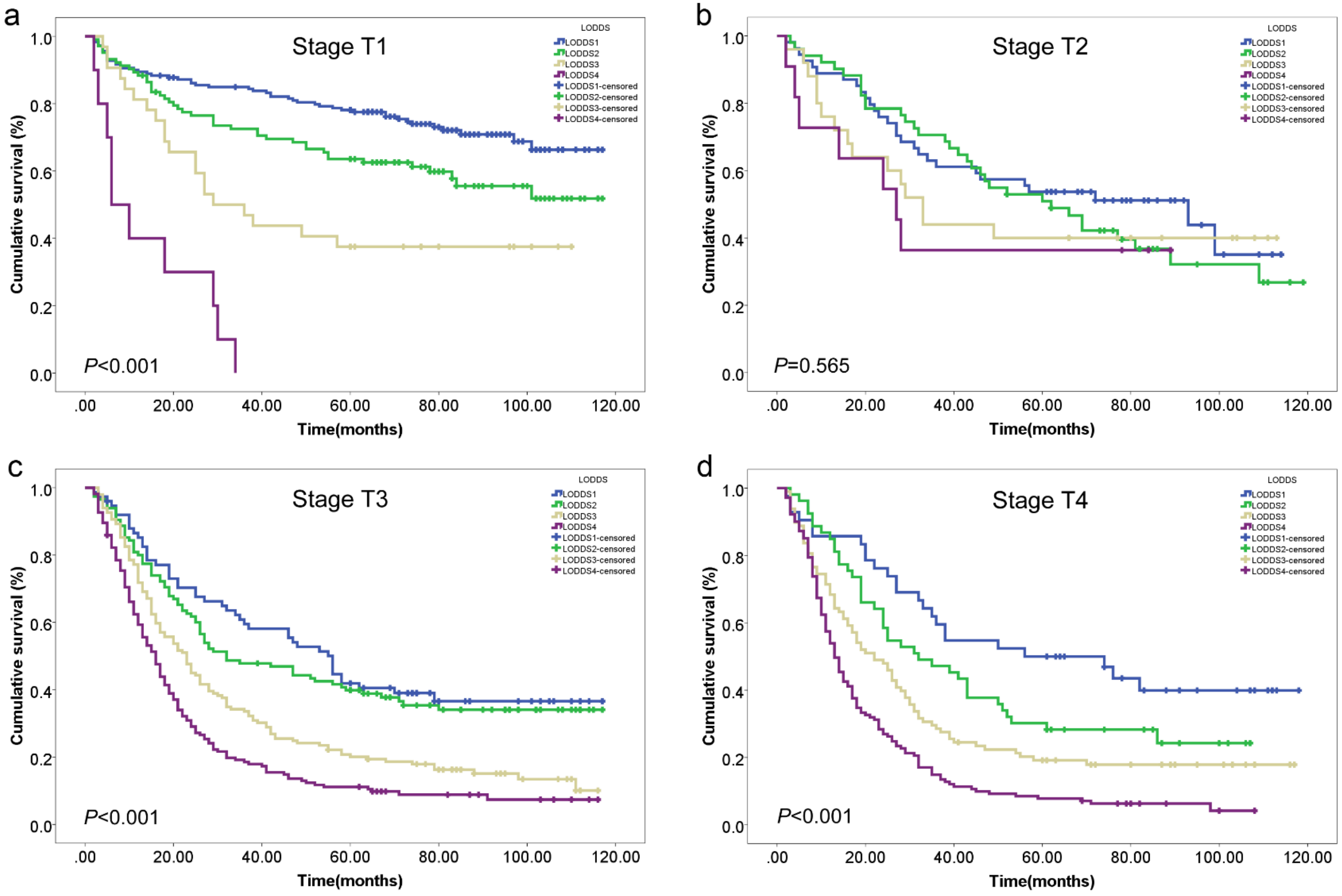

Supplementary Figure 4. Further assessment of the prognostic performance of LODDS in different T stages. The cutoff values of LODDS in different T stages were re-calculated by X-tile analyses. Kaplan-Meier survival curves of LODDS for (a) stage T1 patients (log rank *P*<0.001), (b) stage T2 patients (log rank *P*=0.087), (c) stage T3 patients (log rank *P*<0.001), (d) stage T4 patients (log rank *P*<0.001).

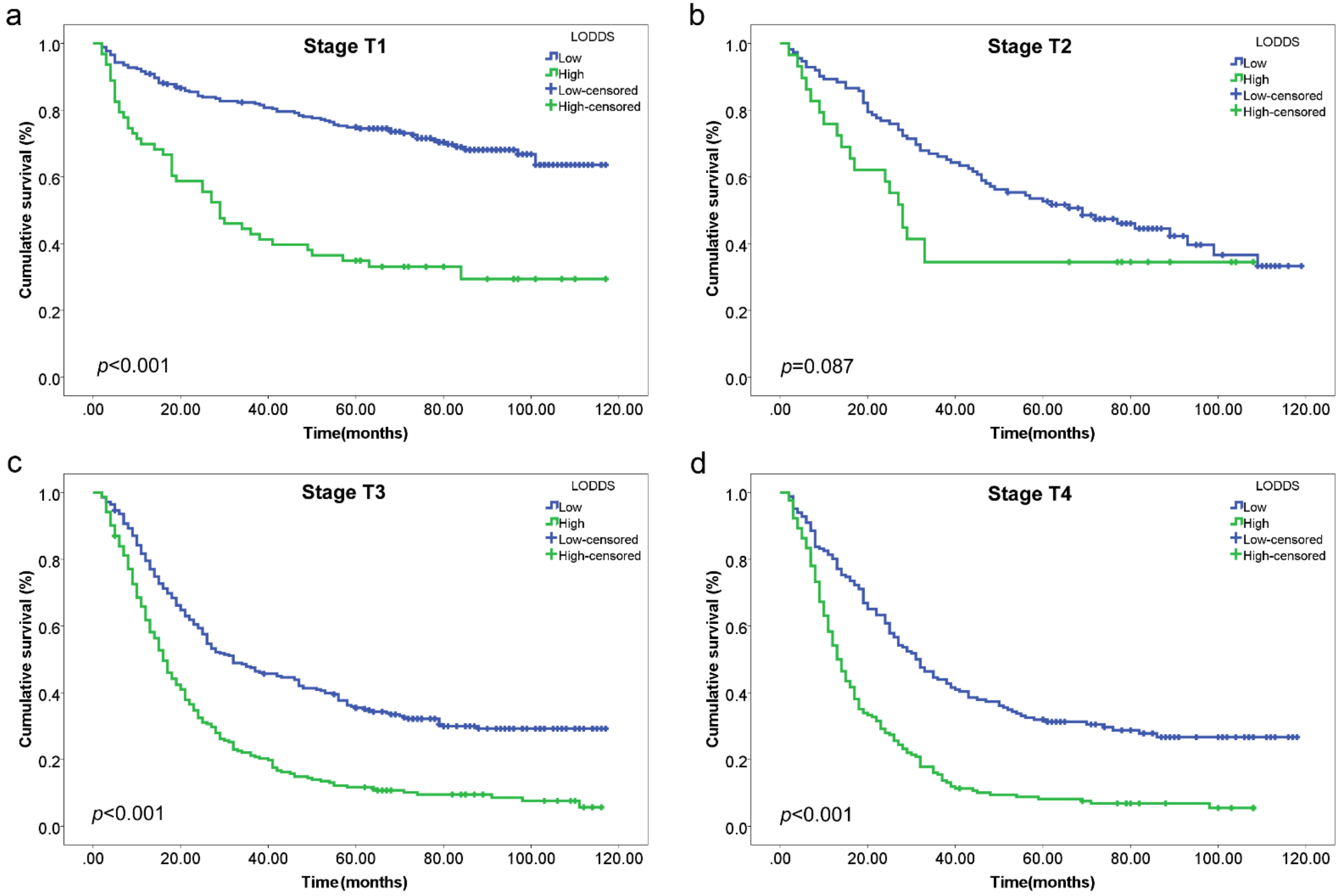

Supplementary Figure 5. Further assessment of the prognostic performance of LODDS in different N stages. The cutoff values of LODDS in different N stages were re-calculated by X-tile analyses. Kaplan-Meier survival curves of LODDS for (a) stage N0 patients (log rank *P*=0.003), (b) stage N1 patients (log rank *P*<0.001), (c) stage N2 patients (log rank *P*<0.001), (d) stage N3 patients (log rank *P*<0.001).

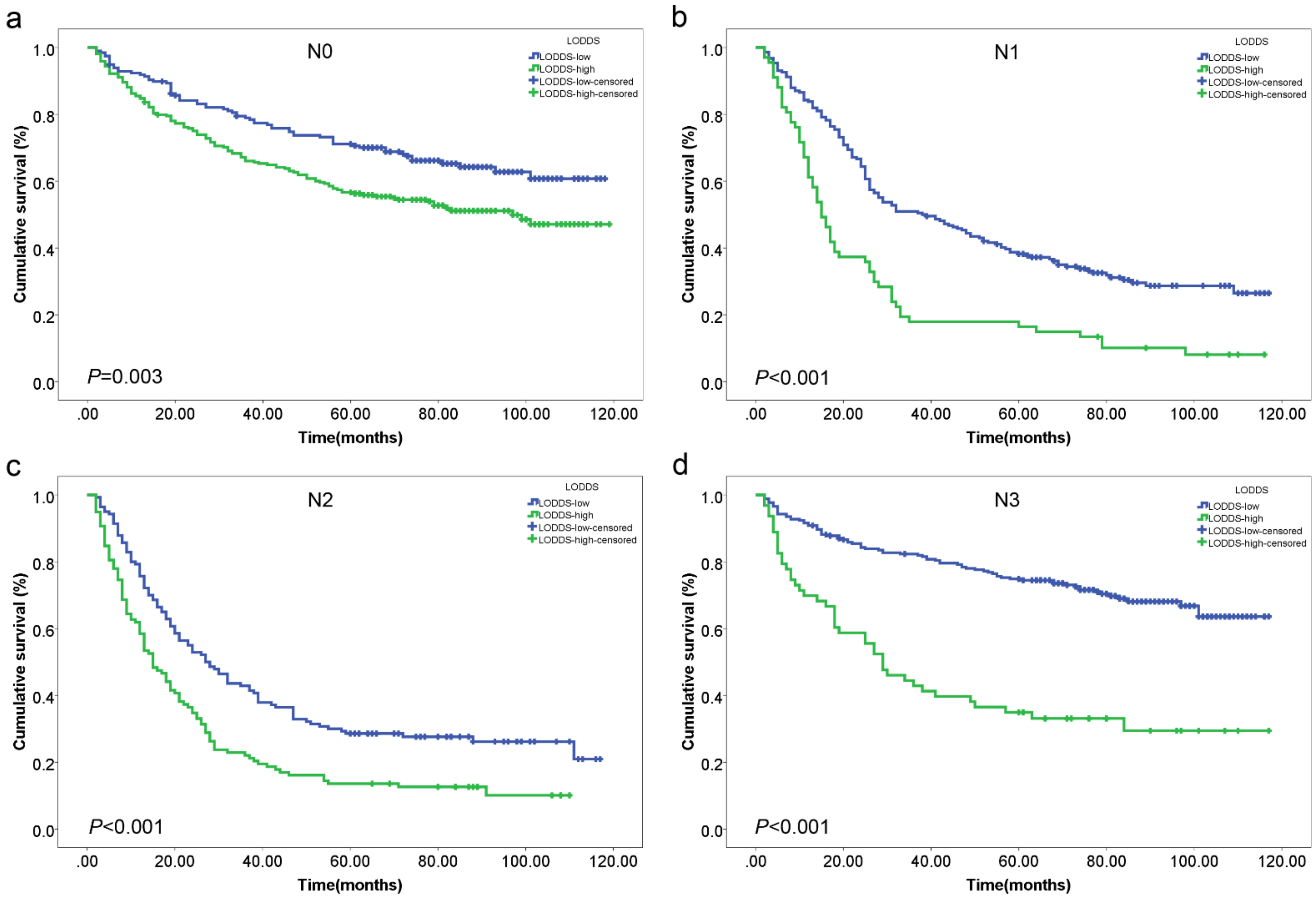

Supplementary Figure 6. Further assessment of the prognostic performance of LODDS in different LNR groups. The cutoff values of LODDS in different LNR groups were re-calculated by X-tile analyses. Kaplan-Meier survival curves of LODDS for (a) stage LNR1 patients (log rank *P*=0.003), (b) stage LNR2 patients (log rank *P*=0.117), (c) stage LNR3 patients (log rank *P*=0.01), (d) stage LNR4 patients (log rank *P*<0.001).

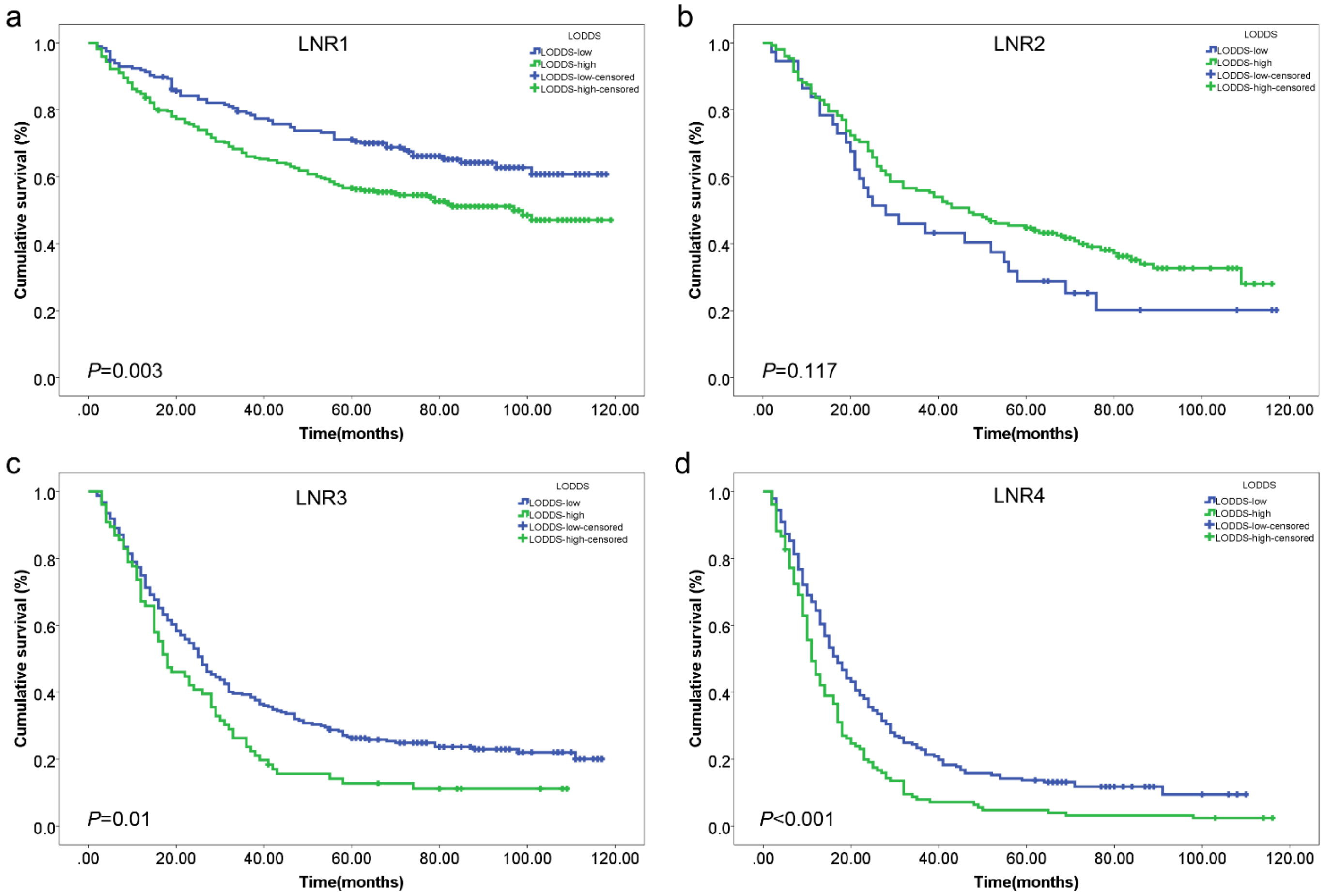

Supplementary Table 1. Prognostic performance of three lymph node staging schemes in different T stages for Siewert type II esophagogastric junction adenocarcinoma.

		Harrell's C-index (95% CI)	AIC	*P*-value1	*P*-value2	Log-rank *P*-value
T1(n=324)	LNM	0.626(0.585-0.667)	440.490	Ref		<0.001
	LNR	0.631(0.590-0.672)	423.370	0.407	Ref	<0.001
	LODDS	0.644(0.593-0.695)	427.050	0.244	0.312	<0.001
T2(n=141)	LNM	0.539(0.472-0.606)	205.400	Ref		0.536
	LNR	0.537(0.469-0.605)	205.850	0.472	Ref	0.552
	LODDS	0.558(0.486-0.630)	205.110	0.282	0.267	0.565
T3(n=502)	LNM	0.580(0.551-0.609)	523.730	Ref		<0.001
	LNR	0.614(0.584-0.644)	502.420	0.016	Ref	<0.001
	LODDS	0.618(0.589-0.647)	494.840	0.008	0.405	<0.001
T4(n=334)	LNM	0.612(0.577-0.647)	287.760	Ref		<0.001
	LNR	0.631(0.597-0.665)	273.010	0.167	Ref	<0.001
	LODDS	0.637(0.602-0.672)	265.910	0.094	0.367	<0.001

AIC: Akaike information criterion

LNM: lymph node metastasis

LNR: positive lymph node ratio

LODDS: log odds of positive lymph node

Ref: reference category

Continuous LNM, LNR and LODDS were included for evaluation.

P-value1 and *P*-value2: C-index comparisons by R language (Pvalue=1-pnorm(abs(r["C X1"]-r["C X2"])/(r["S.D."]/2))).

Log-rank *P*-value: by Mantel-Cox analysis

Supplementary Table 2. Cutoff values of LODDS re-calculated by X-tile analyses in different subgroups and its prognostic performance assessed by log-rank *P* value.

		Sample size	Low interval	No. of patients	High interval	No. of patients	log-rank Chi-square	Log-rank *P*-value
**T stage**	T1	324	(-4.65, -1.90)	261	(-1.90, 3.22)	63	44.861	<0.001
	T2	141	(-5.20, -1.10)	112	(-1.10, 1.61)	29	2.924	0.087
	T3	502	(-4.73, -0.85)	279	(-0.85, 4.04)	223	54.710	<0.001
	T4	334	(-4.39, -0.67)	166	(-0.65, 4.26)	168	47.823	<0.001
**N stage**	N0	466	(-5.20, -3.30)	197	(-3.30, -1.10)	269	8.977	0.003
	N1	283	(-3.34, -1.10)	216	(-1.10, -1.61)	67	25.095	<0.001
	N2	258	(-2.47, -0.75)	140	(-0.75, 2.56)	118	15.938	<0.001
	N3	295	(-1.66, -0.32)	63	(-0.32, 4.26)	232	44.861	<0.001
**LNR category**	LNR1	466	(-5.20, -3.30)	197	(-3.30, -1.10)	269	8.977	0.003
	LNR2	189	(-3.34, -2.65)	37	(-2.65, -1.61)	152	2.459	0.117
	LNR3	323	(-1.83, -0.66)	247	(-0.66, -0.30)	76	6.680	0.010
	LNR4	324	(-0.29, 0.99)	197	(0.99, 4.26)	127	15.668	<0.001

Log-rank *P*-value: by Mantel-Cox analysis

Supplementary Table 3. Linear trend χ2 score to evaluate the discriminatory ability and monotonicity of gradients, and the likelihood ratio (χ2) test to assess homogeneity ability.

	Likelihood Ratio	*P*-value	Linear-by-Linear Association	*P*-value
LNM	263.836	<0.001	106.730	<0.001
LNR	463.304	<0.001	180.901	<0.001
LODDS	591.877	<0.001	207.847	<0.001
